# Smartwatch step counting: impact on daily step-count estimation accuracy

**DOI:** 10.3389/fdgth.2024.1400369

**Published:** 2024-08-08

**Authors:** Peter Düking, Jana Strahler, André Forster, Birgit Wallmann-Sperlich, Billy Sperlich

**Affiliations:** ^1^ Department of Sports Science and Movement Pedagogy, Technische Universität Braunschweig, Braunschweig, Germany; ^2^ Sport Psychology, Deparment of Sport and Sport Science, University of Freiburg, Freiburg im Breisgau, Germany; ^3^ Institute of Psychology, Julius-Maximilians-Universität Würzburg, Wurzburg, Germany; ^4^ Institute of Sport Sciences, Julius-Maximilians-Universität Würzburg, Wurzburg, Germany; ^5^ Integrative and Experimental Exercise Science & Training, Department of Sport Sciences, Julius-Maximilians-Universität Würzburg, Wurzburg, Germany

**Keywords:** innovation, smartwatch, technology, wearable, eHealth, mHealth

## Abstract

**Introduction:**

The effect of displayed step count in smartwatches on the accuracy of daily step-count estimation and the potential underlying psychological factors have not been revealed. The study aimed for the following: (i) To investigate whether the counting and reporting of daily steps by a smartwatch increases the daily step-count estimation accuracy and (ii) to elucidating underlying psychological factors.

**Methods:**

A total of 34 healthy men and women participants wore smartwatches for 4 weeks. In week 1 (baseline), 3 (follow-up 1), and 8 (follow-up 2), the number of smartwatch displayed steps was blinded for each participant. In week 2 (Intervention), the number of steps was not blinded. During baseline and follow-ups 1 and 2, the participants were instructed to estimate their number of steps four times per day. During the 4-week wash-out period between follow-ups 1 and 2, no feedback was provided. The Body Awareness Questionnaire and the Body Responsiveness Questionnaire (BRQ) were used to elucidate the psychological facets of the assumed estimation accuracy.

**Results:**

The mean absolute percentage error between the participants’ steps count estimations and measured steps counts were 29.49% (at baseline), 0.54% (intervention), 11.89% (follow-up 1), and 15.14% (follow-up 2), respectively. There was a significant effect between baseline and follow-up 1 [*t* (61.7) = 3.433, *p *< 0.001] but not between follow-up 1 and follow-up 2 [*t* (60.3) = −0.288, *p* = 0.774]. Only the BRQ subscale “Suppression of Bodily Sensations” appeared to be significant at the Baseline (*p* = 0.012; Bonferroni adjusted *p* = 0.048) as a factor influencing step-count estimation accuracy.

**Conclusion:**

The counting and reporting of daily steps with a smartwatch allows improving the subjective estimation accuracy of daily step counts, with a stabilizing effect for at least 6 weeks. Especially individuals who tend to suppress their bodily sensations are less accurate in their daily step-count estimation before the intervention.

## Introduction

Regular physical activity reduces non-communicable disease prevalence and mortality (1). However, in high-income countries, around 37% of adults did not meet the 2016 World Health Organization (WHO) physical activity guidelines (2). Walking, a common daily activity, serves as a low-threshold intervention to boost daily step count, especially among sedentary individuals (3).

One potential method to encourage people to increase their daily number of steps may derive from electronic health (eHealth) and mobile health (mHealth) (4) solutions employing, e.g., wearable sensors (wearables) such as Smartwatches. Meta-analytical evidence revealed that step-count monitoring using different kinds of wearables (i.e., pedometers, body worn-trackers, and smartphone applications) can increase daily step count in different populations [e.g., community-dwelling adults (5), patients with chronic disease (6), or in school-aged children (7)].

Despite these promising results, other aspects with relevance to the use of wearables are less well understood. For example, there is only little research on the effects of step tracking with wearables on aspects of body awareness. Body awareness can be defined as subjective, phenomenological aspects of proprioception and interoception that enters conscious awareness, which is modifiable by mental processes (e.g., attention or interpretation) (8) and therefore includes step-count estimation accuracy. Here, we define step-count estimation accuracy as how accurately an individual can subjectively estimate the number of steps taken at a certain time during a day. Reporting of step counts is often erroneous (9) and in different scenarios it has been previously argued that enhancing aspects of body awareness is one key element for effectiveness of the different therapeutic approaches (8). While it was argued that engaging in self-monitoring of step counts prompts a process of self-evaluation, potentially resulting in the adaptation of physical activity (10), scarce evidence supporting this argument stems mostly from qualitative research. For example, it was shown that device-based step counting increases the wearer’s conscious awareness regarding steps in different populations (10, 11). However, to the best of our knowledge, there is no quantitative research available that investigates effect of step counting on aspects of body awareness and more specifically on step-count estimation accuracy. In addition, the psychological factors underlying step-count estimation accuracy are not yet fully comprehended, but identifying these factors could assist in determining which individuals can improve step-count estimation accuracy.

The aim of this study was as follows: (i) to investigate the effect of counting and reporting daily steps by a smartwatch on the daily step-count estimation accuracy, and (ii) to elucidate the underlying psychological facets that might explain step-count estimation accuracy.

## Material and methods

### Participants

Altogether 34 healthy participants (16 men, 18 women) of Caucasian origin were informed about all experimental procedures and provided written consent to participate in the study. The study was approved by the institute's ethical committee and was performed following the Declaration of Helsinki. No compensation was given to the participants.

### Smartwatch

A popular end-consumer smartwatch (Forerunner 245, Garmin, Olathe, KS, USA) was used for this study and programmed as indicated by the manufacturer. Previous smartwatch models’ step-count validity at different velocities (4.3, 7.2, 10.1, and 13.0 km h^−1^), intermittent velocities, and while running outdoors at 10.1 km h^−1^ (12) as well as smartwatches by this manufacturer are commonly used in PA research in different populations and settings (13, 14). To further evaluate the validity of smartwatches, three participants wore a smartwatch and a waist-worn triaxial accelerometer (ActiGraph GT3X+, Pensacola, FL, USA) on the hip for 7 days, which was evaluated to have good criterion validity for step counting (15).

### Experimental design

**Figure 1:**
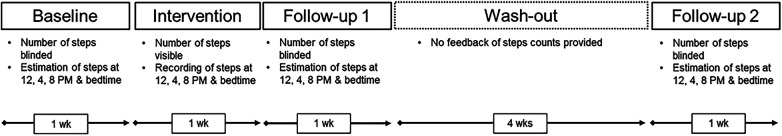
Experimental design.

The experimental design of this study is illustrated in Figure 1.

The participants wore the smartwatches continuously for a total of 4 weeks and were instructed to charge the battery only during the night if needed. In the first week (baseline), third week (follow-up 1), and eighth week (follow-up 2), the numbers of steps provided by the smartwatches were blinded to the participants. Blinding was performed by using a smartwatch-face that did not show daily steps. In week 2 (intervention), the number of steps was not blinded and reported to the participants. During baseline and follow-ups 1 and 2, all participants were instructed to estimate their number of steps four times per day i.e., at 12 PM, 4 PM, 8 PM, and before going to bed. The smartwatch automatically reminded the participants to estimate their achieved number of steps. During the intervention, all were instructed to note their steps in a diary, which was displayed by the smartwatch. A pre-set alarm on the smartwatch reminded the participants each time. Between follow-ups 1 and 2, a wash-out period of 4 weeks was scheduled in which none of the participants wore their smartwatches or any other device for step-count assessment.

### Questionnaires

To assess body awareness, participants were assessed with the Body Awareness Questionnaire (BAQ) and the Body Responsiveness Questionnaire (BRQ) (13). The BAQ measures attentiveness to bodily sensations and processes on a 7-point Likert scale (*not at all true to me* to *very true about me*) and a total score is calculated. The BRQ measures responsiveness to bodily sensations on a 7-point Likert scale with the same anchors as for the BAQ. The results from factor analyses in the BRQ indicated optimal fit by assuming the three subscales “Importance of Interoceptive Awareness,” “Perceived Connection,” and (the single item scale) “Suppression of Bodily Signals.” The German version of both scales that were used in this study showed acceptable internal consistencies (Cronbach's A BAQ = 0.86, BRQ Importance of Interoceptive Awareness = 0.75, BRQ Perceived Connection = 0.75) (16).

### Data preparation and statistical analysis

#### Validity of the used smartwatch to estimate steps

The validity of the smartwatch-derived step count was assessed by calculating Pearson’s r and the mean absolute percentage error (MAPE) between the values provided by the smartwatch and the Actigraph. MAPE was calculated as an average of the absolute difference between the estimation of steps and the smartwatch-measured steps divided by the criterion measure value, multiplied by 100.

#### Assessing difference of estimated steps and smartwatch-measured steps

For each participant, estimations of steps as well as smartwatch-measured steps at bedtime were averaged for baseline, intervention, follow-up 1, and follow-up 2, respectively.

For baseline, intervention, follow-up 1, and follow-up 2, the MAPE as well as Pearson’s r between estimations of steps and smartwatch-measured steps were calculated to provide an indicator of overall error (14) and correlation, respectively. The analysis was performed in Microsoft Excel (Microsoft Corp., Redmond, WA, USA).

**Figure 2:**
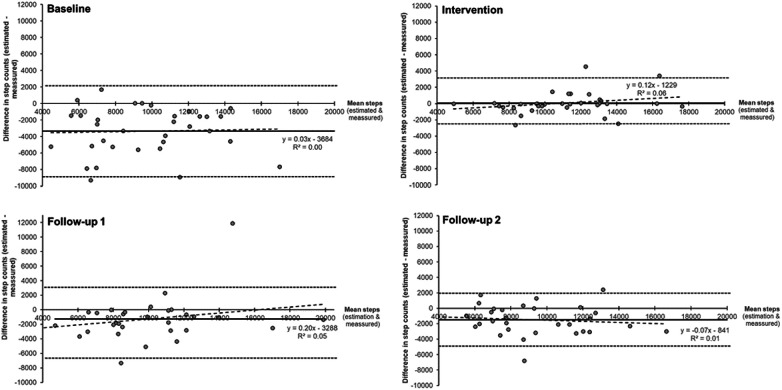
Bland-Altmann plots for estimations of steps and step counts measured by the smartwatch at Baseline, Intervention, Follow-up 1 and 2.

Bland–Altman plots comparing the estimation of steps taken against smartwatch-derived steps are displayed with corresponding 95% limits of agreement at baseline, intervention, follow-up 1, and follow-up 2 (see Figure 2).

A dependent *t*-test assessed the difference between the estimations of steps taken and smartwatch-measured steps taken at baseline, intervention, follow-up 1, and follow-up 2.

To assess the within-person change of daily step-count estimation accuracy, a mixed model analyses with random intercept for each participant was fit in Jamovi (version 2.2.5).

To estimate the overall influence of the smartwatch on daily step-count estimation accuracy, values of the daily step-count estimation and step-count values by the smartwatch were log-transformed (by the natural logarithm) to diminish possible estimation issues arising from skewness, non-normality, or extreme range-differences (ranging from 1,846 to 20,819 steps) within the data. Daily step-count estimation was subtracted from step-count measures by the smartwatch, indexing the discrepancy of both values. The first model included the measurement occasion and step count by the smartwatch as well as their interaction as independent terms. Step count by the watch (log-transformed) was included due to the possibility of greater differences between participant and smartwatch measures resulting solely from changes in activity levels over time (i.e., step-count estimation may be harder when many steps were taken). Measurement occasion was contrast coded to “*repeated*,” comparing follow-up 1 and baseline as well as follow-up 2 and follow-up 1. In summary, the model provided a random intercept for each participant, estimating the dependent variable “step-count difference” (log-transformed, level 1, metric) with fixed effects for “measurement occasion” (ordinal, estimated as a factor in Jamovi, “repeated” contrast coded, level 1), “step count” (metric, grand-mean centered, level 1), and their interaction. No random slopes were included as they would either render the model unidentifiable or result in a singular fit, indicating an absence of relevant interindividual differences. Additional models investigating the influence of psychological variables further included respective questionnaire sum-scores (metric, grand-mean centered, level 2) and their interaction with the measurement occasion as fixed effects (see the following).

An alpha-level of *p* < 0.05 was considered significant.

#### Assessing underlying psychological facets

Grand-mean centered personality facets obtained from the questionnaires were included into the model. Since these are highly correlated with each other, a total of three models were built including one of the BRQ subscales at a time as otherwise significant contributions by a certain facet may be masked by shared variance with other predictors. In these models, especially the interaction between measurement occasion and personality was of interest. Finally, *post hoc* simple effect analyses with personality as the independent variable and measurement occasion as a moderator were conducted to assess effects in more detail. An alpha-level of *p* < 0.05 was considered significant.

## Results

Validity analysis of the smartwatch compared to the Actigraph showed a MAPE of 6.34% and Pearson’s r of 0.98.

Altogether 31 participants (16 men, 15 women, mean age 22.8 ± 1.8 years, body height 173.4 ± 9.8 cm, and body mass 65.5 ± 10.2 kg) completed the full experimental procedure and were included in the analysis.

Three participants could not finish the study owing to personal reasons. Table 1 displays the difference between the estimation of steps and smartwatch-measured steps at bedtime as mean and standard deviation, the results of the significance testing as well as the MAPE for baseline, intervention, follow-up 1, and follow-up 2.

**Table 1:** Difference between the estimation of steps and smartwatch-measured steps at bedtime (mean ± SD), mean absolute percentage error, and Person’s r within the baseline, intervention, follow-up 1 and follow-up 2.

	Difference estimation—Smartwatch, mean ± SD (*p*-value)	Mean absolute percentage error (%)	Pearson’s r
Baseline	−3,346 ± 2,852 (0.0001)	29.5	0.64
Intervention	60 ± 1,362 (0.93)	0.54	0.89
Follow-up 1	−1,258 ± 3,064 (0.19)	11.9	0.68
Follow-up 2	−1,495 ± 1,865 (0.06)	15.1	0.82

Figure 2 displays the Bland–Altman plots for estimations of steps and step counts measured by the smartwatch at baseline, intervention, and follow-ups 1 and 2.

The repeated contrast revealed a significant effect between baseline and follow-up 1 [*t* (61.7) = 3.433, *p *= 0.001] but not between follow-up 1 and follow-up 2 [*t* (60.3) = −0.288, *p *= 0.774] indicating a decrease in the discrepancy between participants’ step estimation accuracy and step counts provided by the smartwatch following the intervention. There is no significant difference between follow-up 1 and follow-up 2, suggesting this effect was stable for 6 weeks. An additional *post-hoc test* also shows significant differences between baseline and follow-up 2: *t* (61.4) = 3.129, *p *= 0.008.

The inclusion of personality facets into the model warranted significant results for one out of three subscales of the BRQ (due to missing data, only 27 participants were analyzed). Table 2 summarizes the interaction effects for each scale.

**Table 2:** Results of fixed effect omnibus tests for personality dimensions of the Body Responsiveness Questionnaire.

Questionnaire	Subscale	*F*	*df* (num)	*df* (den)	*p*	*p* (Bonferroni)
BRQ	Importance of awareness	1.07	2	47.7	0.351	>0.99
	Perceived connection	0.31	2	47.6	0.735	>0.99
	Suppression of bodily sensations	4.85	2	46.1	0.012*	0.048*
BAQ	Not applicable	0.49	2	46.6	0.618	>0.99

BAQ, body awareness questionnaire; *df* (num), degrees of freedom in the numerator; *df* (den), degrees of freedom in the denominator.

*Significant value.

The full models can be reviewed in Supplementary Materials A and B. According to these analyses, “Suppression of Bodily Sensations” significantly predicted step-count estimation accuracy during the baseline [simple effect at baseline: *t* (59.1) = 3.211, *p *= 0.002]. Following the intervention, personality-related differences in step-count estimation accuracy vanished for the duration of 6 weeks as indicated by a significant interaction contrast between baseline and follow-up 1 and suppression of bodily sensations [*t* (45.2) = 2.383, *p *= 0.021], on the one hand, and a highly insignificant interaction contrast for follow-up 1 to follow-up 2 and suppression of bodily sensations, on the other [*t* (47.3) = −0.483, *p *= 0.631].

## Discussion

The aim of this study was as follows: (i) to investigate the effect of counting and reporting daily steps by a smartwatch on the daily step-count estimation accuracy, and (ii) to elucidate the underlying psychological facets that might explain step-count estimation accuracy.

The main findings of the present experiment are as follows:

(i) The mean absolute percentage error between the estimation of steps and smartwatch-measured steps decreased from baseline (29.49%) to follow-up 1 (11.89%) and increased in follow-up 2 (15.14%). Strengthening these results is the repeated contrast analysis, which revealed a significant effect between baseline and follow-up 1, but not between follow-up 1 and follow-up 2 indicating a decrease in the discrepancy between participants’ step estimation accuracy and the step counts provided by the smartwatches following the intervention, which seems to be a stable effect for at least 6 weeks.
(ii) Especially individuals who tend to suppress their bodily sensations are less accurate in their daily step-count estimation prior to the intervention.
(iii) The effect of the investigated personality characteristics on step-count estimation accuracy vanished after the intervention, lasting for at least 6 weeks.

The results are in line with currently available qualitative research findings. Thorup et al. showed that feedback on steps provided by pedometers supported the awareness of walking activity for cardiac patients and health professionals (11). Similarly, McCormack et al. showed that wearing pedometers that record daily step counts increases awareness of how much time is spent being active each day and can motivate people to move more (10). Adding to this literature, the results of the present study quantify for the first time the improvement of step-count estimation accuracy following an intervention period of 1 week and following a wash-out period using a quantitative research design. This study showed that an intervention period of 1 week is sufficient to reduce error of step-count estimation accuracy, which seems to be a stable effect for at least 6 weeks in healthy individuals in the given setting. This shows that with a comparably short intervention period, aspects of body awareness (i.e., step-count estimation accuracy) can be improved, which arguably is a key element to promote physical activity behavior (8, 10). To further strengthen evidence in this direction, future studies should investigate the interaction of body awareness (e.g., step-count estimation accuracy) and measures of physical activity (e.g., step counts).

This study assessed underlying psychological facets that could explain the improvement in step-count estimation accuracy based on questionnaire data and found evidence for the negative effect of “suppression of bodily sensations” on the accuracy of step-count estimation at baseline, but not for “subjective importance of interoceptive awareness “(i.e., appreciating somatic signals as a source of valuable information), the “perceived connection to bodily sensations” (i.e., feeling that bodily signals may oppose current motivational tendencies), or “body awareness” in general (i.e., being sensitive to signals of fatigue or hunger; measured via the BAQ). This observation suggests that the misestimation of step count may not stem from a lack of attentiveness to bodily signals, but rather from suppression of readily available bodily information. Future studies might use this information and screen individuals with particular focus on suppression of bodily sensations prior to an intervention that might be indicative of which individuals can estimate their step-count accurately. In this context, future research needs to investigate if interventions targeting reduction of suppression of bodily sensations can lead to an increase in step-count estimation accuracy.

### Strengths, limitations, and suggestions for future research

The strengths of our study include its longitudinal design with a follow-up test 6 weeks after the intervention to assess whether effects of the intervention are acute or persistent as well as the assessment of underlying personality facets that could account for effects of our intervention.

The results are limited to the effects of step counting using a smartwatch on step-count estimation accuracy in healthy male and female participants. Future studies should investigate the effect in more individuals and other, less active populations, such as sedentary, clinical, or rehabilitative populations, to validate our findings. Also, future research with larger sample sizes should investigate the effect of step counting with smartwatches on step count accuracy, particularly in individuals who tend to suppress their bodily sensations. In addition, while this study showed that estimation in step-count accuracy can be improved by the herein-employed intervention, future research should assess if an increase in estimation in step-count accuracy results in an increase in accumulated daily steps.

## Conclusions

This study demonstrated that counting and reporting daily steps with a smartwatch improves the subjective estimation accuracy of daily step counts, with a stabilizing effect lasting for at least 6 weeks. Especially individuals who tend to suppress their bodily sensations are less accurate in their daily step count estimation before the intervention.

## Data Availability

The raw data supporting the conclusions of this article will be made available by the authors upon reasonable request.
