# Supplementary material for: Smartwatch step counting: impact on daily step-count estimation accuracy
Source: Front Digit Health. 2024 Aug 8;6:1400369. doi: 10.3389/fdgth.2024.1400369 (PMC11339027; doi:10.3389/fdgth.2024.1400369)
Supplement: Supplementary file 1 [file Datasheet1.docx]

**Appendix A: Fixed Omnibus tests for the subscales of the Body responsiveness questionnaire and the Body Awareness Questionnaire**

| **BRQ Subscale: Perceived Connection** | | | | |
| --- | --- | --- | --- | --- |
|  | F | Num df | Den df | p |
| Count by watch (log-transformed) | 0,8626 | 1 | 64,3 | 0,356 |
| Watch-Based Intervention | 7,0815 | 2 | 46,8 | 0,002 |
| Perceived Connection | 3,3889 | 1 | 26,7 | 0,077 |
| Count by watch (log-transformed) ✻ Watch-Based Intervention | 0,0353 | 2 | 56,7 | 0,965 |
| Watch-Based Intervention ✻ Perceived Connection | 0,31 | 2 | 47,6 | 0,735 |
| **BRQ Subscale: Suppression of bodily signals** | | | | |
|  | F | Num df | Den df | p |
| Count by watch (log-transformed) | 0,0461 | 1 | 66,3 | 0,831 |
| Watch-Based Intervention | 9,4246 | 2 | 46,1 | < .001 |
| Suppression | 2,0548 | 1 | 25 | 0,164 |
| Count by watch (log-transformed) ✻ Watch-Based Intervention | 0,1889 | 2 | 53,1 | 0,828 |
| Watch-Based Intervention ✻ Suppression | 4,8536 | 2 | 46,1 | 0,012 |
| Note. Satterthwaite method for degrees of freedom |  |  |  |  |
| **BRQ Subscale: Importance of Awareness** | | | | |
|  | F | Num df | Den df | p |
| Watch-Based Intervention | 7,6998 | 2 | 46,6 | 0,001 |
| Count by watch (log-transformed) | 0,0813 | 1 | 63,9 | 0,776 |
| Importance of Awareness | 0,2322 | 1 | 25,8 | 0,634 |
| Watch-Based Intervention ✻ Count by watch (log-transformed) | 0,0403 | 2 | 54,2 | 0,961 |
| Watch-Based Intervention ✻ Importance of Awareness | 1,0707 | 2 | 47,7 | 0,351 |
| Note. Satterthwaite method for degrees of freedom |  |  |  |  |
| **Body Awareness Questionnaire** | | | | |
|  | F | Num df | Den df | p |
| Watch-Based Intervention | 7,00592 | 2 | 46,4 | 0,002 |
| Count by watch (log-transformed) | 0,31843 | 1 | 65,3 | 0,574 |
| BAQ | 0,01265 | 1 | 25,2 | 0,911 |
| Watch-Based Intervention ✻ Count by watch (log-transformed) | 0,00524 | 2 | 55 | 0,995 |
| Watch-Based Intervention ✻ BAQ | 0,48564 | 2 | 46,6 | 0,618 |
| Note. Satterthwaite method for degrees of freedom |  |  |  |  |

**Appendix B: Full Model analyzing the effects of personality facets affecting accuracy in step count estimation**

| Names | Effect | Estimate | SE | 95% Confidence Interval (lower limit) | 95% Confidence Interval (upper limit) | df | t | p |
| --- | --- | --- | --- | --- | --- | --- | --- | --- |
| (Intercept) | (Intercept) | 0,2934 | 0,048 | 0,1994 | 0,387 | 24,4 | 6,115 | < .001 |
| Count by watch (log-transformed) | Count by watch (log-transformed) | 0,0253 | 0,1178 | -0,2056 | 0,256 | 66,3 | 0,215 | 0,831 |
| Watch-Based Intervention1 | Baseline - POST1 | 0,2727 | 0,0795 | 0,1169 | 0,429 | 47 | 3,431 | 0,001 |
| Watch-Based Intervention2 | POST1 - POST2 | 0,0438 | 0,0793 | -0,1117 | 0,199 | 45,4 | 0,552 | 0,584 |
| Suppression | Suppression | 0,0495 | 0,0345 | -0,0182 | 0,117 | 25 | 1,433 | 0,164 |
| Count by watch (log-transformed) ✻ Watch-Based Intervention1 | Count by watch (log-transformed) ✻ Baseline - POST1 | -0,1483 | 0,2431 | -0,6248 | 0,328 | 52,8 | -0,61 | 0,544 |
| Count by watch (log-transformed) ✻ Watch-Based Intervention2 | Count by watch (log-transformed) ✻ POST1 - POST2 | 0,0479 | 0,2354 | -0,4135 | 0,509 | 53,1 | 0,203 | 0,84 |
| Watch-Based Intervention1 ✻ Suppression | Baseline - POST1 ✻ Suppression | 0,1356 | 0,0569 | 0,0241 | 0,247 | 45,2 | 2,383 | 0,021 |
| Watch-Based Intervention2 ✻ Suppression | POST1 - POST2 ✻ Suppression | 0,0282 | 0,0584 | -0,0863 | 0,143 | 47,3 | 0,483 | 0,631 |
